# Wafer-Level Highly Dense Metallic Nanopillar-Enabled High-Performance SERS Substrates for Molecular Detection

**DOI:** 10.3390/nano13111733

**Published:** 2023-05-25

**Authors:** Pei Zeng, Mengjie Zheng, Hao Chen, Guanying Chen, Zhiwen Shu, Lei Chen, Huikang Liang, Yuting Zhou, Qian Zhao, Huigao Duan

**Affiliations:** 1 State Key Laboratory of Tribology in Advanced Equipment, Department of Mechanical Engineering, Tsinghua University, Beijing 100084, China; zengpei@tsinghua.edu.cn; 2 Jihua Laboratory, Foshan 528000, China; 3 College of Mechanical and Vehicle Engineering, National Engineering Research Centre for High Efficiency Grinding, Hunan University, Changsha 410082, China; 4 Greater Bay Area Innovation Institute, Hunan University, Guangzhou 511300, China; 5 Tsinghua Shenzhen International Graduate School, Tsinghua University, Beijing 100084, China

**Keywords:** SERS, hot spots, large-scale production, sensitivity, flexible substrate

## Abstract

Seeking sensitive, large-scale, and low-cost substrates is highly important for practical applications of surface-enhanced Raman scattering (SERS) technology. Noble metallic plasmonic nanostructures with dense hot spots are considered an effective construction to enable sensitive, uniform, and stable SERS performance and thus have attracted wide attention in recent years. In this work, we reported a simple fabrication method to achieve wafer-scale ultradense tilted and staggered plasmonic metallic nanopillars filled with numerous nanogaps (hot spots). By adjusting the etching time of the PMMA (polymethyl methacrylate) layer, the optimal SERS substrate with the densest metallic nanopillars was obtained, which possessed a detection limit down to 10^−13^ M by using crystal violet as the detected molecules and exhibited excellent reproducibility and long-term stability. Furthermore, the proposed fabrication approach was further used to prepare flexible substrates; for example, a SERS flexible substrate was proven to be an ideal platform for analyzing low-concentration pesticide residues on curved fruit surfaces with significantly enhanced sensitivity. This type of SERS substrate possesses potential in real-life applications as low-cost and high-performance sensors.

## 1. Introduction

Surface-enhanced Raman spectroscopy (SERS) is one of the most effective methods to realize the molecular detection of ultrasmall amounts [1], thus having wide significant applications in chemical analysis [2,3], bioimaging [4,5], environmental monitoring [6,7], and food security [8,9,10,11]. The large electromagnetic (EM) field enhancement phenomenon supported by localized surface plasmon resonance (LSPR) excited by the incident light has been verified to be the most important factor in the SERS effect. A promising SERS substrate should contain numerous highly dense hot spots, where the large field enhancement is produced. Extensive research has been conducted in the development of high-performance SERS substrates since the discovery of SERS in the 1970s. It has been well proven that hot spots play a key role in SERS [12,13,14], which can produce surface plasmon resonance, dramatically enhancing the Raman signal of the detected molecule. To realize the possible maximal SERS enhancement factor (EF), it is of great importance to produce as many possible appropriate plasmonic hot spots to support the near-field enhancement at the plasmon resonance condition. Dense noble metallic nanostructures, such as gold and silver, have been confirmed to be the most effective construction to generate hot spots [15,16,17]. Exploring facile means to reliably obtain plasmonic metallic nanostructures with as many hot spots as possible has attracted much attention in recent years in the SERS community.

There are currently two main technologies to prepare metallic plasmonic nanoparticles, including top–down fabrication or bottom–up self-assembly processes [18,19,20,21,22]. The bottom–up processes provide the advantages of low cost, facilitating, and extremely high sensitivity by arranging hot spots. However, the reliable assembly of plasmonic nanostructures into uniform distribution to meet the requirements of reproducible detection is still challenging. The hot spots commonly cannot be controllably prepared and homogeneously formed. In contrast, the top–down methods based on lithographic techniques can support precise control of the geometries, dimensions, and arrangements of the fabricated nanostructures. Furthermore, the top–down methods are in principle CMOS compatible and are promising for batch fabrication for SERS-based applications. For example, nanoimprint lithography provided an excellent approach to obtaining large-area plasmonic nanostructures with high throughput [23]. However, this method produced plasmonic nanostructures with a limited density, only moderately enhancing the plasmonic response. Dorpe et al. demonstrated a scalable and uniform SERS substrate with ultradense nanogap-enabled hot spots [24]. However, it involved extravagant ultraviolet (DUV) immersion lithography. Expensive lithography procedures are usually indispensable in top–down methods, resulting in dramatically increased costs. The above-mentioned top–down methods based on lithographic techniques are usually unable to achieve dense hot spots over a large area at a low cost. Therefore, the reliable low-cost fabrication of uniform, reproducible, and stable wafer-level SERS substrates with high sensitivity is still a big challenge.

In this paper, we proposed and demonstrated a simple nanolithography-free fabrication approach to generate ultradense metallic tilted and staggered plasmonic nanopillars over a 4-inch wafer. Actually, the area of the proposed SERS substrate was only limited by the sizes of the holders in our etcher and evaporator facility. Numerous metallic nanogaps embedded in the dense staggered metallic nanopillars can provide a large number of high-density hot spots to produce huge electric field enhancement for SERS detection. This nanolithography-free SERS substrate with dense metallic nanopillars showed a highly uniform, sensitive, and stable performance. The density of metallic nanopillars can be adjusted by the reactive ion etching time. The lowest detectable limit for crystal violet molecules on the optimal substrate was proven to be only 10^−13^ M. Furthermore, the proposed fabrication approach was further applied to prepare flexible SERS substrates, and the obtained flexible SERS substrate can simply test 10^−4^ M chlorpyrifos pesticides on the curved surface of an apple. The proposed SERS substrate with ultradense hot spots can be used as a promising configuration to push SERS for practical applications.

## 2. Experimental Sections

### 2.1. Materials

All of the reagents were of analytical grade and used as received. Si (n-type, 1–10 Ω·cm) substrate was purchased from Suzhou Yancai Micro-nano Technology Co., Ltd. PMMA (polymethyl methacrylate, 4% 950 k PMMA in anisole) was purchased from MicroChem Corp. Crystal violet was purchased from Sinopharm Chemical Reagent Co., Ltd. Chlorpyrifos solution in acetonitrile was purchased from Aladdin Industrial Co., Ltd. Silver particles used for thermal evaporation were purchased from ZhongNuo Advanced Material (Beijing) Technology Co., Ltd. The flexible PDMS (polydimethylsiloxane) substrate was fabricated referring to this work [25].

### 2.2. Fabrication of SERS Substrates

**Figure 1:**
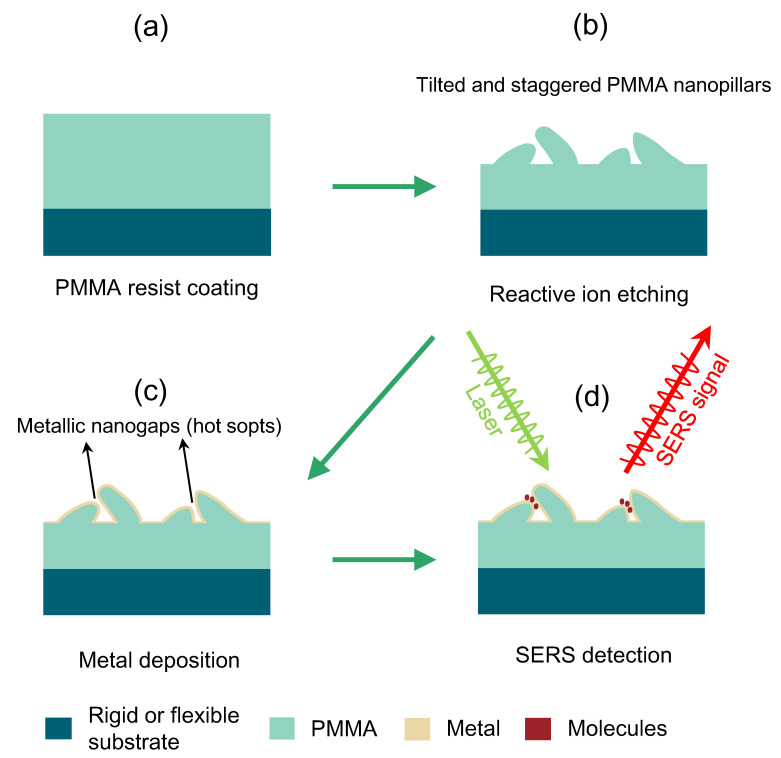
Schematic illustration of the fabrication of the proposed SERS substrate. (**a**) PMMA layer coating. (**b**) Oxygen reactive ion etching. (**c**) Metal deposition of silver. (**d**) Molecular adsorption and Raman detection.

A brief flow chart of the proposed process for obtaining SERS substrate is shown in Figure 1. First, a 230 nm thick PMMA (950 K) film was spin-coated on the substrate and baked on a hot plate at 180 °C for 3 min (Figure 1a). Subsequently, the samples were then quickly transferred into the reactive ion etching equipment (Etchlab-300, Zhejiang Service Vacuum & Optics Technology Co., Ltd.) to etch the PMMA layer (Figure 1b). The used gas was oxygen, the gas flow rate was 160 sccm, the pressure during the etching process was 2 Pa, and the radio frequency power was 100 W. It is worth noting that the grassy tilted and staggered PMMA nanopillar surface can be formed on the substrate due to the sufficient pressure in etched regions after the etching process [26]. The density of residual PMMA nanopillars can be easily controlled by the etching time. Afterward, the metallization of the samples was performed in a thermal evaporation system (JSD300, Anhui Jiashuo Vacuum Technology Co. Ltd.) (Figure 1c). The working pressure was kept at <4 × 10^−4^ Pa. The Ag deposition rate was kept at about 1 Å·s^−1^. The thickness of all evaporated silver film in this work was 30 nm, which was monitored by an angstrom-sensitive quartz-crystal microbalance. Finally, the samples were subjected to SERS measurement (Figure 1d).

### 2.3. Characterization

The digital photos were obtained with an iPhone 12. The observation of metallic nanopatterns was performed by a field-emission scanning electron microscope (SEM) (Sigma HD VP, Carl Zeiss) with an accelerating voltage of 10 kV. X-ray diffraction (XRD) patterns were collected using the D8 ADVANCE diffractometer (AXS GmbH, Bruker). The thickness of the PMMA layer was measured by a SE-VE spectroscopic ellipsometer (Wuhaneoptics Technology Co., Ltd.). The surface topography of the samples was conducted by an atomic force microscope (AFM, Dimension Icon, Bruker). The optical reflection spectra of the samples were obtained by using a spectrometer (NOVA, Ideaoptics). The strain–stress curves were measured using a mechanical testing apparatus (ZQ-990A, China) with a 20 N load cell.

### 2.4. SERS Measurement

For detecting crystal violet molecules, the crystal violet standard solution was prepared by dissolving the crystal violet powder into deionized water. All samples were incubated in the crystal violet solution dissolved in DI water for 24 h and then dried with nitrogen gas to obtain an average single molecular layer [27,28]. After that, the samples were subjected to SERS measurement.

For detecting chlorpyrifos pesticide residues, 0.5 mL of 10^−4^ M chlorpyrifos solution in acetonitrile was dropped on the surface of an apple. The flexible SERS substrate was then used to tightly wipe the apple surface to extract the chlorpyrifos residues. After the evaporation of the solvent at room temperature, the sample was subjected to SERS measurement.

The SERS detection was performed using a WITec Alpha 300R confocal Raman microscopy system by using a 532 nm laser excitation. The SERS signals were collected by a ×50 (0.75 N.A.) microscope objective lens under a laser power intensity of 2 mW with an integration time of 2 s for three times.

### 2.5. FDTD Simulation

**Figure 2:**
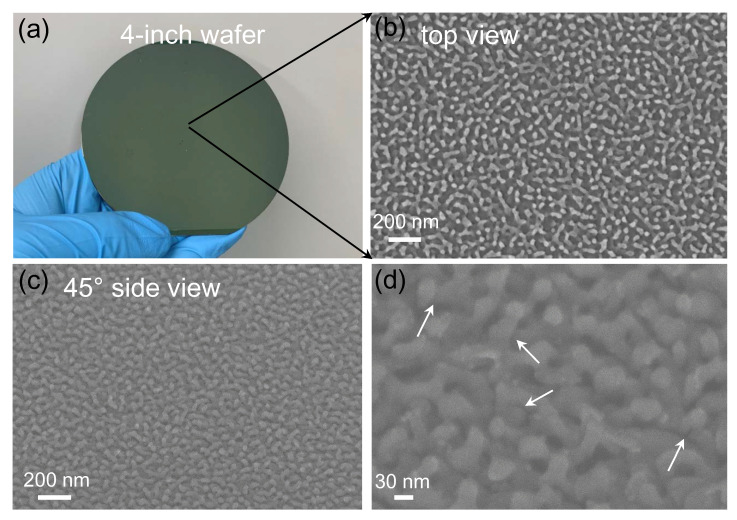
The digital and SEM images of the proposed SERS substrate. (**a**) A digital image of the fabricated 4-inch wafer SERS substrate. (**b**–**d**) SEM images of the ultradense grassy tilted and staggered metallic nanopillar clusters: (**b**) top view and (**c**,**d**) 45° side view.

The simulations were conducted by the three-dimensional FDTD solutions (Lumerical Solutions, Version 8.15.736) to obtain the electric field distribution profile [17,27]. The simplified calculated model based on Figure 2d was used for a simple qualitative analysis of electromagnetic field enhancement. When modeling the nanopillar SERS structure, the diameter of PMMA nanopillars was set as 10 nm, and the diameter of Ag-coated nanopillars was set as 20 nm, and the nanogap was defined as 2 nm. A total-field scattered field source was used with the polarized electric field along the x-axis. The dispersion models of Ag and Si were all obtained from the database of Palik, while the used refractive indices for the PMMA were 1.46. A grid size of 1 nm and perfectly matched layer boundary conditions in all three directions were used in the simulation.

## 3. Results and Discussion

The proposed method involves no complicated or costly fabrication processes and is inherently appropriate for obtaining large-scale substrates as expounded in Section 2.2. Indeed, the SERS sample is on a 4-inch Si wafer (Figure 2a), only limited by the sizes of the sample stages in the etcher and evaporator facility. Figure 2b shows a top-view SEM image of the dense grassy metallic nanopillar clusters. Most of the PMMA nanopillars were broken and collapsed during the PMMA etching process, resulting in a distinguishable tilted and staggered nanopillar surface. It should be noted that the morphology of the PMMA layer was proved to be unchanged after metal deposition [3], and this type of grassy surface topography is quite effective for immobilizing molecules and providing numerous hot spots for SERS detection [26]. Figure 2c,d shows the 45° side-view SEM images, revealing the dense tilted nanopillar surface topography and numerous metallic nanogaps embedded in the staggered metallic nanopillars (some of these nanogaps are marked by the arrows in Figure 2d). These numerous metallic nanogaps can support huge electric field enhancement for SERS detection.

**Figure 3:**
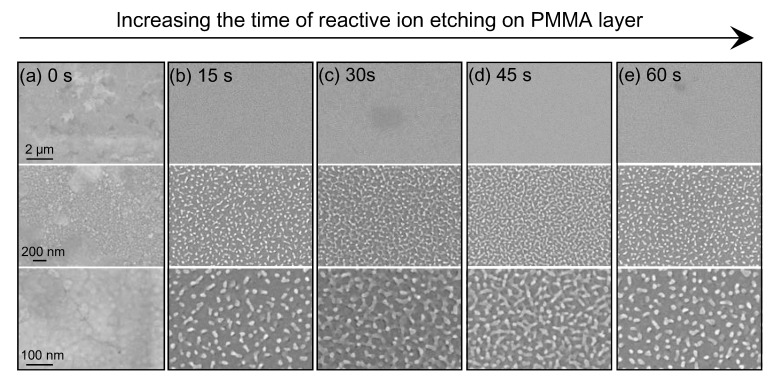
SEM images with varying magnifications of the SERS substrates fabricated by different times of reactive ion etching: (**a**) 0 s, (**b**) 15 s, (**c**) 30 s, (**d**) 45 s, and (**e**) 60 s.

The compositions of the PMMA substrates before and after etching were characterized by XRD, and the representative patterns are shown in Figure S1. The representative diffraction peaks of the PMMA substrates before and after etch did not vary obviously, suggesting that the crystal structures of the PMMA nanopillar clusters remained unchanged. By precisely varying the etch time of the PMMA layer, tilted and staggered nanopillar clusters of different densities can be formed. As shown in Figure 3, with the increase in etching time from 0 to 45 s, the thickness of the PMMA layer decreased and PMMA nanopillars grew denser, resulting in the consequent increase in hot spots embedded in Ag nanopillars. When the etching time was more than 45 s, the PMMA layer tended to be completely etched (the functional relationship between the remaining PMMA thickness and the etching time was given in Figure S2), and some PMMA nanopillars were gradually removed by etching, resulting in the formation of sparse Ag nanopillars on the substrate. Therefore, the densities of Ag nanopillars on the SERS substrate can be easily regulated by adjusting the etching time. Further evidence of AFM characterization on these densities of Ag nanopillars is given in Figure S3. It indicated that the densest metallic nanopillars were formed with an etching time of 45 s. Figure S4 shows the reflectance spectra for SERS substrates fabricated by various etching times from 0 to 60 s. It can be seen that the reflectance dip blue-shifted with decreased PMMA layer thickness. It indicated that the coupling between the Ag layer and Si substrate increased with their aggregation, leading to a blueshift of the plasmon resonance. This is consistent with previous theoretical research [29,30].

**Figure 4:**
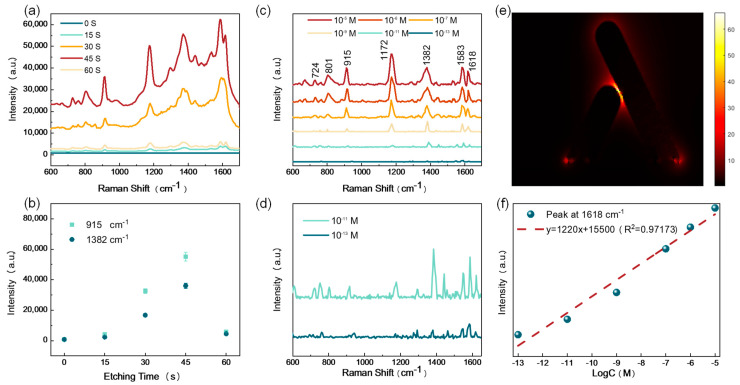
(**a**) SERS signals of CV molecules on SERS substrates prepared by various times of reactive ion etching. (**b**) The statistical plot of SERS peak intensities of CV molecules at 915 and 1382 cm^−1^ on SERS substrates prepared by various times of reactive ion etching. (**c**) SERS spectra of various CV concentrations on the optimal SERS substrate. (**d**) Zoomed-in SERS spectra of CV molecules at concentrations of 10^−11^ M and 10^−13^ M. (**e**) Simplified electric field simulation of tilted and staggered metallic nanopillars. (**f**) Logarithmic plot of Raman intensity at 1618 cm^−1^ as a function of CV concentrations.

Crystal violet (CV) molecules were chosen as the detected molecule to experimentally test the SERS activity of these metallic nanopillar cluster-based substrates. Figure 4a shows the measured SERS spectra of 10^−5^ M CV from the SERS substrates obtained by various etching times. It can be seen from Figure 4b that the intensities of Raman signals increased with increased etching times at first. When the etching time of the PMMA layer reached 45 s, the optimal SERS substrate was obtained. The densest tilted and staggered nanopillars were obtained at an etching time of 45 s, as shown in Figure 3d, contributing to the densest effective hot spots among the layer metallic nanopillars, which can support better efficiency and more activity for SERS measurement. When the etching time was longer than 45 s, nanopillars became sparse and tended to vanish, which obviously decreased the effective hot spots. Therefore, the SERS intensity from the substrate with an etching time of 60 s became weaker. As discussed above, the densest Ag nanopillars obtained with an etching time of 45 s generated the highest number of hot spots and produced the strongest SERS signals. Hence, the optimal SERS substrates obtained by an etching time of 45 s acted as the samples used in the experiments that followed.

The ultralow concentration detection is of great importance for SERS substrates, which exhibit outstanding SERS sensitivity. To further investigate the capability of the proposed SERS substrate, the low-concentration detection test of the optimal SERS substrate was conducted with various concentrations of CV molecules from 10^−5^ to 10^−13^ M. To eliminate the effect of possible residual CV molecules, the substrate was repeatedly rinsed using deionized water after each measurement. Figure 4c shows the baseline-removed Raman spectra of CV molecules with various concentrations from 10^−5^ to 10^−13^ M. All of the main Raman characteristic peaks can be identified distinctly for CV concentrations down to 10^−9^ M, showing that the proposed SERS substrate has a high-sensitivity SERS detection ability. To further analyze the SERS signals of CV concentration from 10^−11^ and 10^−13^ M, the zoomed-in spectra are given in Figure 4d. It can be seen that the characteristic peaks can still be distinguished, even though the concentration of CV is as low as 10^−13^ M. The simplified calculated electric field distribution of the nanopillar structure under the 532 nm laser excitation is shown in Figure 4e (XZ cross-sectional plane). The used simplified calculated model for a simple qualitative analysis was constructed based on Figure 2d. The strongest electric field enhancement exists in the nanogap region between two adjacent metallic nanopillars, verifying the importance of numerous metallic nanogaps embedded in these staggered nanopillars in high-sensitivity SERS detection. Figure 4d demonstrates the high-degree linear relationship between the Raman peak intensity at 1618 cm^−1^ and the concentration of CV solution. The best-fitting linear regression equation is as follows: y = 1220x + 15,500, where x refers to the logarithmic CV concentration (correlation coefficient = 0.97173). The essentially linear change of SERS intensity as a function of molecule concentration manifests that the proposed substrate can be available for the quantitative analysis of trace substances in practical use.

The SERS enhancement factor (EF) is an important parameter to quantitatively characterize the SERS substrate, although the definition of EF is highly varied. The definition of the SERS EF [31,32] and the details of the calculation process in this work are described in the Support Information. The baseline-removed Raman spectrum of the reference capillary tube sample is shown in Figure S5. The final calculated value of the SERS EF was 2.02 × 10^6^. This EF of 2.02 × 10^6^ is comparable to a lot of reported high-performance SERS substrates. For example, Wang et al. demonstrated an EF of 1.5 × 10^6^ using the particle-on-film structure [31]. Additionally, Gong et al. showed that their sensitive Q-tip-based SERS substrate had an EF of 1.6 × 10^6^ [32]. Furthermore, this EF of 2.02 × 10^6^ is not high as that of self-assembled metallic nanostructures with tiny nanogaps, but this proposed “top–down” process-enabled substrate ought to have better SERS uniformity and reproducibility.

**Figure 5:**
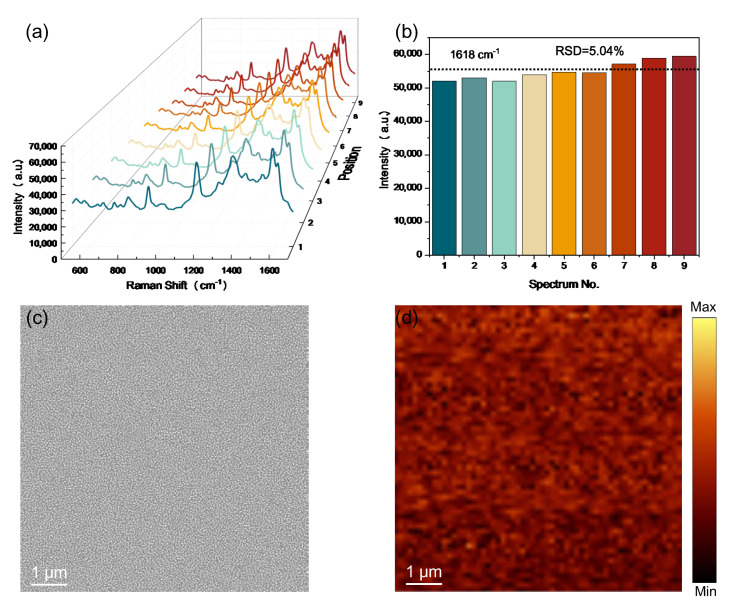
The uniformity of the proposed SERS substrate. (**a**) SERS spectra of CV molecules from nine randomly selected spots on the obtained SERS substrate. (**b**) The recorded Raman signal intensities of CV molecules at 1618 cm^−1^ peak. (**c**) SEM image of the optimal SERS substrate with the large-area uniform surface morphology. (**d**) Spatial SERS mapping with a 532 nm laser exciting the 1172 cm^−1^ crystal violet Raman peak across Figure 5c.

The detection uniformity and reproducibility of SERS signals from the substrate are crucial for practical use. To further study the detection uniformity and reproducibility of the proposed SERS substrate, the SERS spectra of a CV concentration of 10^−5^ M were recorded from nine randomly selected spots over the optimal SERS substrate under the same measurement conditions, as shown in Figure 5a. The corresponding intensity variation of the nine characteristic peaks at 1618 cm^−1^ is further given in Figure 5b to demonstrate the uniform property of the proposed SERS substrate. The statistic on the average SERS intensity of different sites showed a small deviation of 5.04%, revealing that the optimal SERS substrate possessed excellent reproducibility over the whole substrate surface. This is due to the uniform surface morphology of the optimal SERS substrate obtained by the proposed “top–down” processes. An SEM image of large-area uniform surface morphology is given in Figure 5c. Figure 5d shows the spatial SERS mapping with a 532 nm laser exciting the 1172 cm^−1^ crystal violet Raman peak across Figure 5c, further confirming the uniform property of the proposed SERS substrate.

**Figure 6:**
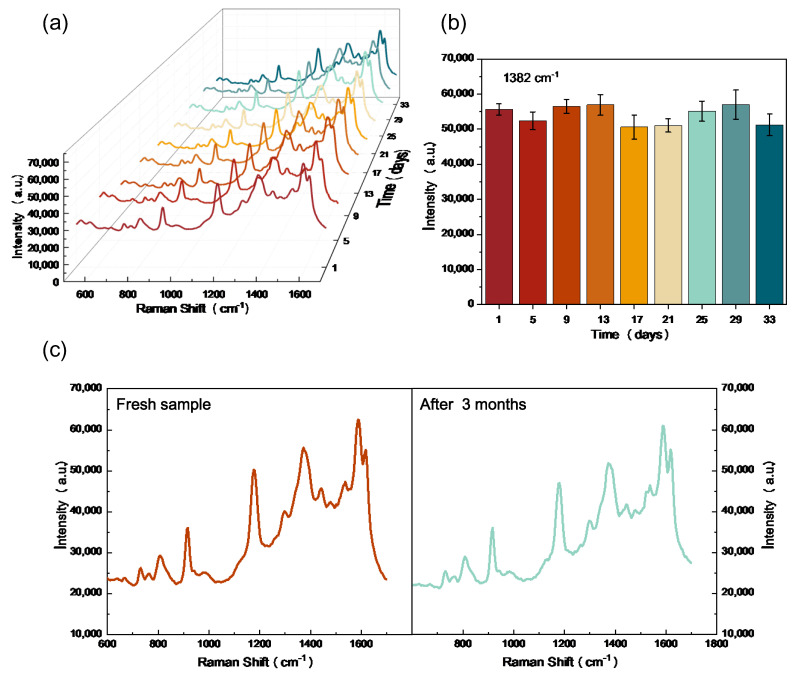
The long-term stability test of the proposed SERS substrate. (**a**) SERS spectra of CV molecules from the substrate during 33 days. (**b**) The recorded Raman signal intensities of CV molecules at 1382 cm^−1^ peak during 33 days. (**c**) Raman spectra of fresh SERS sample and SERS sample after storage for 3 months.

The test stability is also a highly important property to evaluate the SERS substrates. To investigate the long-term stability of the proposed SERS substrate. Raman spectra of a CV concentration of 10^−5^ M were collected every 4 days over the duration of 34 days at room temperature. The measured SERS spectra are displayed in Figure 6a, demonstrating that the proposed substrate can maintain its fine features and intensities. There is only a small alteration in the average SERS intensity over a period of 34 days, as shown in Figure 6b, which can mainly be owing to the slight disturbances in measuring environments. The long-term stability of the SERS substrate was further assessed by comparing the SERS signals from the freshly prepared sample and the sample stored for 3 months, as shown in Figure 6c. Overall, the intensity values showed excellent reproducibility. The long-term test stability supports the proposed substrate to be a promising SERS sensing platform in long-life applications.

**Figure 7:**
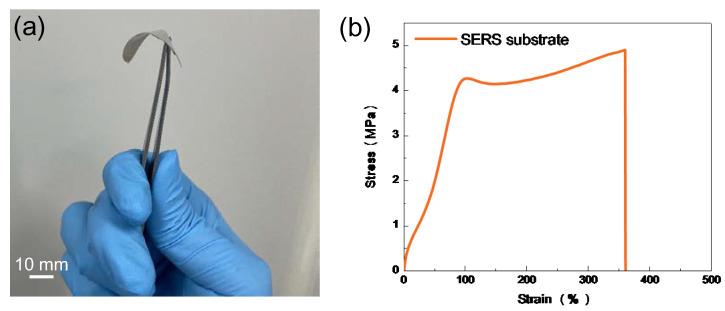

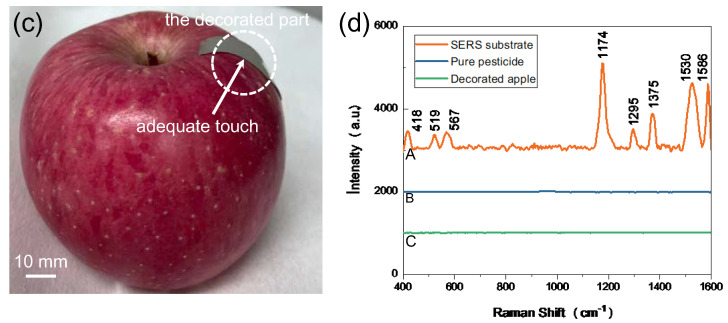
Detection of the pesticide residues on an apple surface using flexible PMDS-based SERS substrates. (**a**) Digital photo of the fabricated flexible SERS substrate. (**b**) Tensile strain tests of the fabricated flexible SERS substrate. (**c**) Digital photo of collecting chlorpyrifos molecules by bonding the flexible SERS substrate and the apple. (**d**) The recorded Raman spectra of a chlorpyrifos concentration of 10^−4^ M.

SERS has been regarded as an effective detective technique for the analysis of pesticide residues in agricultural products. Chlorpyrifos is a type of extensively used organophosphate insecticide in agriculture and urban areas for pest control. However, chlorpyrifos pesticide residues may bring risks to human health. Compared with the traditional rigid SERS substrate, flexible SERS substrates can absorb the pesticide molecules on irregular and curved fruit or vegetable surfaces, enabling on-site detection of residual pesticide analytes without additional extraction steps. The proposed process in this work is also applicable to soft and flexible SERS substrates. PDMS material was used as the flexible supporter for the fabricated flexible SERS substrate due to its advantages of high elasticity, high ductility, chemical stability, and cost-effectiveness. The flexible PDMS substrate was fabricated referring to this literature [25]. Figure 7a displays the obtained PMDS-based SERS substrate by the fabrication process described in Figure 1. Figure 7b shows the measured tensile strain test results of the fabricated PMDS-based SERS substrate, demonstrating that the substrate can be stretched to over 350% compared with its original length. It indicated that the PMDS-based SERS substrate possesses excellent mechanical performance and has the potential to be used in various extreme conditions. Due to the excellent mechanical properties of the PDMS substrate, the SERS substrate can adequately touch the decorated apple surface to extract the pesticide residues, as shown in Figure 7c. The SERS measurement results are shown in Figure 7d. Spectra “A–C” represent the Raman signals of a chlorpyrifos concentration of 10^−4^ M collected from the flexible SERS substrate, the pure chlorpyrifos pesticide, and the pesticide-decorated apple surface, respectively. No characteristic SERS peak can be clearly distinguished from the pure chlorpyrifos pesticide or the pesticide-moistened apple surface, while all of the representative SERS peaks of chlorpyrifos can be identified clearly from the fabricated flexible SERS substrate. All the detected characteristic SERS peaks are basically consistent with the reported SERS spectra of chlorpyrifos molecules. According to the reported literature [33,34,35], tentative vibration mode assignments for chlorpyrifos molecules were as follows. The peak at 418 cm^−1^ can be assigned to P–O–C stretching vibration, the peak at 519 cm^−1^ was related to P–O stretching vibration, the peak at 567 cm^−1^ was associated with P=S or C–Cl stretching vibration, the peak at 1174 cm^−1^ may be attributed to ring breathings, the peak at 1295 cm^−1^ may be associated with Cl-ring vibration, the peaks at 1375 cm^−1^ and 1530 cm^−1^ may be all related to Cl-ring vibration, and the peak at 1586 cm^−1^ can be assigned to ring stretching. It indicated that the proposed flexible platform has high-performance SERS detection capacity, and the proposed strategy is a valid method for the on-site test of pesticide residues.

## 4. Conclusions

In summary, we proposed and demonstrated a nanolithography-free method to realize a wafer-scale SERS substrate with ultradense tilted and staggered metallic nanopillars that generated a highly sensitive, uniform, and reproducible SERS response. The density of Ag nanopillars and the SERS signal intensity of the SERS substrate can be regulated by the reactive ion etching time of the PMMA layer. When the etching time was 45 s, the optimal densest plasmonic nanopillar-enabled SERS substrate was achieved, which possessed excellent uniformity (about 5%), long-term stability (over 3 months), and high SERS sensitivity to CV with a detectable concentration as low as 10^−13^ M. The proposed approach was further extended to prepare PDMS-based flexible SERS substrates. With its excellent mechanical flexibility, the flexible SERS substrate can be used to simply test 10^−4^ M chlorpyrifos on the curved surface of an apple. Due to its ease of preparation, low cost, wafer scale, and high SERS performance, we believe that the proposed approach is promising for the detection of ultrasmall amounts of substances in label-free sensing applications, propelling SERS for practical applications in chemical and biological analysis, environment monitoring, and the food safety field.

## Data Availability

All data that support the findings of this study are included within the article (and any Supplementary Files).

## Supplementary Materials

The following supporting information can be downloaded at: https://www.mdpi.com/article/10.3390/nano13111733/s1, Figure S1. XRD patterns of the PMMA substrate before and after reactive ion etching. Figure S2. The functional relationship between the remaining PMMA thickness and the etching time. Figure S3. Atomic force microscopy characterization of the SERS substrates obtained by different times of reactive ion etching: (a) 15 s, (b) 30 s, (c) 45 s, and (d) 60 s. Figure S4. Reflection spectra of the SERS substrates obtained by different times of reactive ion etching. Figure S5. The baseline-removed Raman spectrum of the reference capillary tube sample with 10^−1^ M CV concentration.

## References

[1] Nie S., Emory S.R. (1997). Probing Single Molecules and Single Nanoparticles by Surface-Enhanced Raman Scattering. Science.

[2] Cao Y.C., Jin R., Mirkin C.A. (2002). Nanoparticles with Raman Spectroscopic Fingerprints for DNA and RNA Detection. Science.

[3] Zheng M., Chen Y., Liu Z., Liu Y., Wang Y., Liu P., Liu Q., Bi K., Shu Z., Zhang Y. (2019). Kirigami-inspired multiscale patterning of metallic structures via predefined nanotrench templates. Microsyst. Nanoeng..

[4] Zavaleta C.L., Smith B.R., Walton I., Doering W., Davis G., Shojaei B., Natan M.J., Gambhir S.S. (2009). Multiplexed imaging of surface enhanced Raman scattering nanotags in living mice using noninvasive Raman spectroscopy. Proc. Natl. Acad. Sci. USA.

[5] Andreou C., Neuschmelting V., Tschaharganeh D.-F., Huang C.-H., Oseledchyk A., Iacono P., Karabeber H., Colen R.R., Mannelli L., Lowe S.W. (2016). Imaging of Liver Tumors Using Surface-Enhanced Raman Scattering Nanoparticles. ACS Nano.

[6] Wagner M., Lin K.-Y.A., Oh W.-D., Lisak G. (2021). Metal-organic frameworks for pesticidal persistent organic pollutants detection and adsorption—A mini review. J. Hazard. Mater..

[7] Xu J., Huang Y., Zhang S., Liu Z., Jiang S. (2023). Plasmon-induced hot carrier separation across multicomponent heterostructure in Ag@AgCl@g-C3N4 composites for recyclable detection-removal of organic pollutions via SERS sensing. Appl. Surf. Sci..

[8] Liu B., Han G., Zhang Z., Liu R., Jiang C., Wang S., Han M.-Y. (2012). Shell Thickness-Dependent Raman Enhancement for Rapid Identification and Detection of Pesticide Residues at Fruit Peels. Anal. Chem..

[9] Han L., Liu H., Zhang J., Zhou J., Jiang T. (2022). Recyclable SERS monitoring of food quality based on the shrubby morphology of titania oxide-triggered electromagnetic “hotspots”. Appl. Surf. Sci..

[10] Chen Y., Ge F., Guang S., Cai Z. (2018). Low-cost and large-scale flexible SERS-cotton fabric as a wipe substrate for surface trace analysis. Appl. Surf. Sci..

[11] Augustine S., Sooraj K., Pachchigar V., Krishna C.M., Ranjan M. (2021). SERS based detection of Dichlorvos pesticide using silver nanoparticles arrays: Influence of array wavelength/amplitude. Appl. Surf. Sci..

[12] Lim D.-K., Jeon K.-S., Hwang J.-H., Kim H., Kwon S., Suh Y.D., Nam J.-M. (2011). Highly uniform and reproducible surface-enhanced Raman scattering from DNA-tailorable nanoparticles with 1-nm interior gap. Nat. Nanotechnol..

[13] Kang M., Park S.-G., Jeong K.-H. (2015). Repeated Solid-state Dewetting of Thin Gold Films for Nanogap-rich Plasmonic Nanopillars. Sci. Rep..

[14] Chirumamilla M., Toma A., Gopalakrishnan A., Das G., Zaccaria R.P., Krahne R., Rondanina E., Leoncini M., Liberale C., De Angelis F. (2014). 3D Nanostar Dimers with a Sub-10-nm Gap for Single-/Few- Molecule Surface-Enhanced Raman Scattering. Adv. Mater..

[15] Taylor R.W., Coulston R.J., Biedermann F., Mahajan S., Baumberg J.J., Scherman O.A. (2013). In Situ SERS Monitoring of Photochemistry within a Nanojunction Reactor. Nano Lett..

[16] Tian S., Neumann O., McClain M.J., Yang X., Zhou L., Zhang C., Nordlander P., Halas N.J. (2017). Aluminum Nanocrystals: A Sustainable Substrate for Quantitative SERS-Based DNA Detection. Nano Lett..

[17] Zheng M., Zhu X., Chen Y., Xiang Q., Duan H. (2017). Three-dimensional donut-like gold nanorings with multiple hot spots for surface-enhanced raman spectroscopy. Nanotechnology.

[18] Chung T., Koker T., Pinaud F. (2016). Split-GFP: SERS Enhancers in Plasmonic Nanocluster Probes. Small.

[19] Oh Y.-J., Jeong K.-H. (2012). Glass Nanopillar Arrays with Nanogap-Rich Silver Nanoislands for Highly Intense Surface Enhanced Raman Scattering. Adv. Mater..

[20] Si S., Liang W., Sun Y., Huang J., Ma W., Liang Z., Bao Q., Jiang L. (2016). Facile Fabrication of High-Density Sub-1-nm Gaps from Au Nanoparticle Monolayers as Reproducible SERS Substrates. Adv. Funct. Mater..

[21] Cai H., Wu Y., Dai Y., Pan N., Tian Y., Luo Y., Wang X. (2016). Wafer scale fabrication of highly dense and uniform array of sub-5 nm nanogaps for surface enhanced Raman scatting substrates. Opt. Express.

[22] Zheng M., Yang Y., Zhu D., Chen Y., Shu Z., Berggren K.K., Soljačić M., Duan H. (2021). Enhancing Plasmonic Spectral Tunability with Anomalous Material Dispersion. Nano Lett..

[23] Ou F.S., Hu M., Naumov I., Kim A., Wu W., Bratkovsky A.M., Li X., Williams R.S., Li Z. (2011). Hot-Spot Engineering in Polygonal Nanofinger Assemblies for Surface Enhanced Raman Spectroscopy. Nano Lett..

[24] Li J., Chen C., Jans H., Xu X., Verellen N., Vos I., Okumura Y., Moshchalkov V.V., Lagae L., Van Dorpe P. (2014). 300 mm Wafer-level, ultra-dense arrays of Au-capped nanopillars with sub-10 nm gaps as reliable SERS substrates. Nanoscale.

[25] Kim G.-M., Lee S.-J., Kim C.-L. (2021). Assessment of the Physical, Mechanical, and Tribological Properties of PDMS Thin Films Based on Different Curing Conditions. Materials.

[26] Nabesawa H., Hitobo T., Wakabayashi S., Asaji T., Abe T., Seki M. (2008). Polymer surface morphology control by reactive ion etching for microfluidic devices. Sens. Actuators B Chem..

[27] Zeng P., Shu Z., Zhang S., Liang H., Zhou Y., Ba D., Feng Z., Zheng M., Wu J., Chen Y. (2021). Fabrication of single-nanometer metallic gaps via spontaneous nanoscale dewetting. Nanotechnology.

[28] Zeng P., Liu Q., Zheng M., Chen Y., Liu G., Duan H. (2020). Ion-beam-etching based lift-off for reliable patterning of dense and inverse metallic nanostructures towards 10-nm scale. Microelectron. Eng..

[29] Lévêque G., Martin O.J. (2006). Optical interactions in a plasmonic particle coupled to a metallic film. Opt. Express.

[30] Lévêque G., Martin O.J. (2006). Tunable composite nanoparticle for plasmonics. Opt. Lett..

[31] Wang X., Zhu X., Chen Y., Zheng M., Xiang Q., Tang Z., Zhang G., Duan H. (2017). Sensitive Surface-Enhanced Raman Scattering Detection Using On-Demand Postassembled Particle-on-Film Structure. ACS Appl. Mater. Interfaces.

[32] Gong Z., Du H., Cheng F., Wang C., Wang C., Fan M. (2014). Fabrication of SERS Swab for Direct Detection of Trace Explosives in Fingerprints. ACS Appl. Mater. Interfaces.

[33] Shende C., Inscore F., Sengupta A., Stuart J., Farquharson S. (2010). Rapid extraction and detection of trace Chlorpyrifos-methyl in orange juice by surface-enhanced Raman spectroscopy. Sens. Instrum. Food Qual. Saf..

[34] Ma P., Wang L., Xu L., Li J., Zhang X., Chen H. (2020). Rapid quantitative determination of chlorpyrifos pesticide residues in tomatoes by surface-enhanced Raman spectroscopy. Eur. Food Res. Technol..

[35] Xu Q., Guo X., Xu L., Ying Y., Wu Y., Wen Y., Yang H. (2017). Template-free synthesis of SERS-active gold nanopopcorn for rapid detection of chlorpyrifos residues. Sens. Actuators B Chem..

