# Supplementary material for: Wafer-Level Highly Dense Metallic Nanopillar-Enabled High-Performance SERS Substrates for Molecular Detection
Source: Nanomaterials (Basel). 2023 May 25;13(11):1733. doi: 10.3390/nano13111733 (PMC10254535; doi:10.3390/nano13111733)
Supplement: Supplementary file 1 [file nanomaterials-13-01733-s001.zip › nanomaterials-2425235-supplementary.pdf]

Support Information For

Wafer-Level Highly Dense Metallic Nanopillar-Enabled High-Performance SERS Substrates for Molecular Detection

Pei Zeng [1,†], Mengjie Zheng [2,*,†], Hao Chen [2], Guanying Chen [2], Zhiwen Shu [3,4], Lei Chen [3,4], Huikang Liang [3,4], Yuting Zhou [5], Qian Zhao [1,*] and Huigao Duan [3,4]

[1] State Key Laboratory of Tribology in Advanced Equipment, Department of Mechanical Engineering, Tsinghua University, Beijing 100084, China
[2] Jihua Laboratory, Foshan 528000, China
[3] College of Mechanical and Vehicle Engineering, National Engineering Research Centre for High Efficiency Grinding, Hunan University, Changsha 410082, China
[4] Greater Bay Area Innovation Institute, Hunan University, Guangzhou 511300, China
[5] Tsinghua Shenzhen International Graduate School, Tsinghua University, Beijing 100084, China
[*] Correspondence: zhengmj@jihualab.ac.cn (M.Z.); zhaoqian@tsinghua.edu.cn (Q.Z.)
[†] These authors contribute equally to this work.

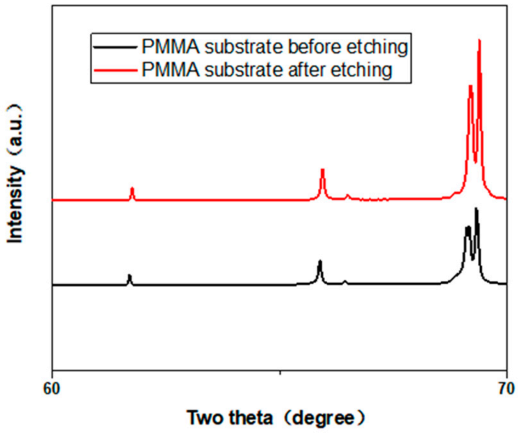

Figure S1. XRD patterns of the PMMA substrate before and after reactive ion etching.

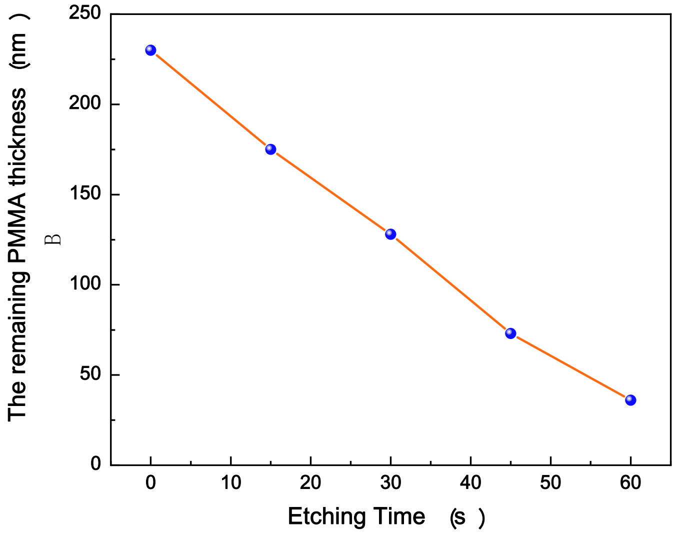

Figure S2. The functional relationship between the remaining PMMA thickness and the etching time.

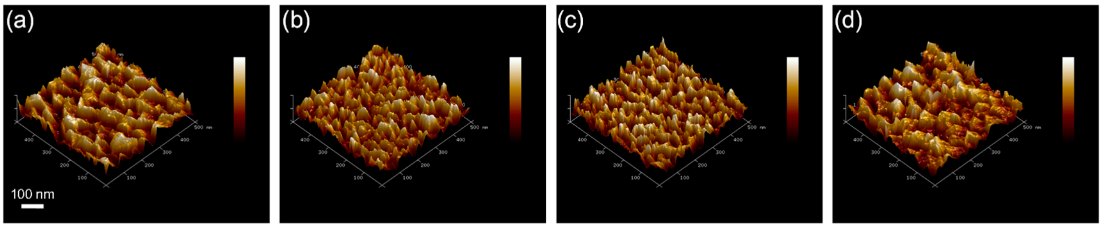

Figure S3. Atomic force microscopy characterization of the SERS substrates obtained

by different times of reactive ion etching: (a) 15 s, (b) 30 s, (c) 45 s, and (d) 60 s. It indicated the densest Ag nanopillars were obtained with an etching time of 45 s.

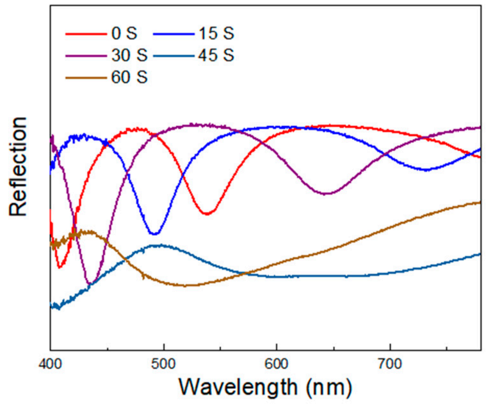

Figure S4. Reflection spectra of the SERS substrates obtained by different times of reactive ion etching.

The details of the SERS EF calculation

The corresponding SERS enhancement factor was assessed by applying the formula:

$$EF = \frac{I_{sers}/N_{sers}}{I_{sample}/N_{sample}}$$

where I_{SERS} and I_{sample} are the measured SERS intensities at the 1618 cm^{-1} Raman peak of the detected CV molecules from the baseline-removed Raman spectra using a Gaussian–Lorentzian fitting for the proposed SERS substrate and the reference capillary tube sample, respectively. N_{SERS} and N_{sample} are the numbers of the CV molecules attached to the surface of the proposed substrate and the reference capillary tube sample, respectively. N_{SERS} can be expressed as

$$N_{sers} = \frac{\pi r^2}{\sigma}$$

where r is the semidiameter of the laser spot (~2 μm), and σ is the occupied area of a single-molecule (~4 nm^2). N_{sample} can be expressed as

$$N_{sample} = \pi r^2 hcN_A$$

where c is the concentration of reference CV molecules (10^{-1} M), h is the confocal depth of the illuminated laser, and N_A is the Avogadro constant.

The baseline-removed Raman spectrum of the reference capillary tube sample was as follows:

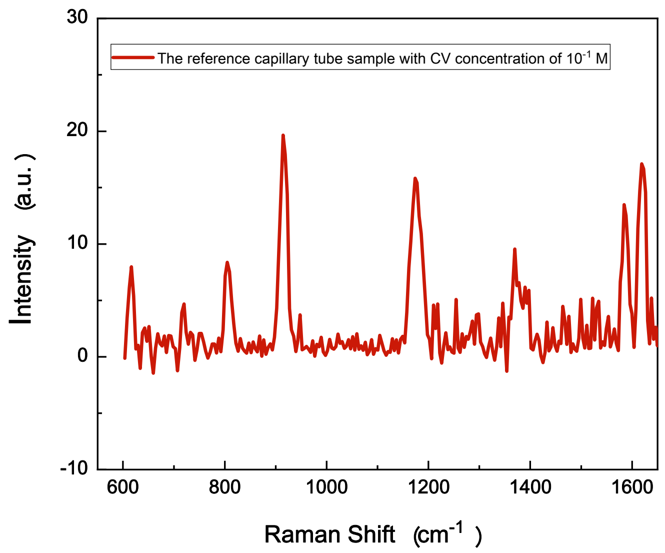

Figure S5. The baseline-removed Raman spectrum of the reference capillary tube sample with 10^{-1} M CV concentration.
